# The GT1-TPS Structural Domain Protein From *Haemonchus contortus* Could Be Suppressive Antigen of Goat PBMCs

**DOI:** 10.3389/fimmu.2021.787091

**Published:** 2022-01-04

**Authors:** Zhaohai Wen, Muhammad Tahir Aleem, Kalibixiati Aimulajiang, Cheng Chen, Meng Liang, Xiaokai Song, Lixin Xu, Xiangrui Li, Ruofeng Yan

**Affiliations:** ^1^Ministry of Education (MOE) Joint International Research Laboratory of Animal Health and Food Safety, College of Veterinary Medicine, Nanjing Agricultural University, Nanjing, China; ^2^State Key Laboratory of Pathogenesis, Prevention and Treatment of High Incidence Diseases in Central Asia, Xinjiang Medical University, Urumqi, China

**Keywords:** *Haemonchus contortus*, trehalose phosphate synthase, cytokines, PBMCs, immunomodulation

## Abstract

Trehalose phosphate synthase (TPS), a key enzyme in trehalose synthesis, is not present in mammals but critical to the viability of a wide range of lower organisms. However, almost nothing is known about the function of Hc-TPS (GT1-TPS structural domain protein from *Haemonchus contortus*). In this study, Hc-TPS gene was cloned and the recombinant protein (rHc-TPS) was expressed and purified. The quantitative real-time PCR (qPCR) results showed that Hc-TPS was transcribed at different stages of *H. contortus*, with higher levels of transcription at the molting and embryo stages. Immunofluorescence analysis showed that Hc-TPS was widely distributed in adults, but the expression was mainly localized on the mucosal surface of the intestine as well as in the embryos of female worms. The impacts of rHc-TPS on peripheral blood mononuclear cell (PBMC) proliferation, nitric oxide (NO) generation, transcriptional expression of cytokines, and related pathways were examined by co-incubating rHc-TPS with goat PBMCs. The results showed that rHc-TPS significantly inhibited PBMC proliferation and NO secretion in a dose-dependent manner. We also found that rHc-TPS activated the interleukin (IL)-10/signal transducer and activator of transcription 3/suppressor of cytokine signaling 3 (IL-10/STAT3/SOCS3) axis and significantly promoted SOCS3 expression, while inhibiting interferon-gamma (INF-γ), IL-4, IL-9, and IL-2 pathways. Our findings may contribute to understanding the immune evasion mechanism for the parasite during host–parasite interactions and also help to provide ideas for discovering new drug targets.

## Introduction

*H. contortus* is an important pathogenic nematode causing huge economic losses worldwide (1) and is able to coexist with its host under harsh conditions that escape digestion by stomach acid and pepsin. The main control measures for haemonchosis are based on the use of anthelmintics. However, the excessive and uncontrolled use of some antiparasitic drugs has led to widespread parasite resistance over the decades (2). *H. contortus* is characterized by genetic diversity and a multistage life cycle (3, 4), which helps the worm to evade the host’s immune system and also contributes to the development of drug-resistant strains (5). Therefore, an in-depth study of key molecules involved in the mechanism of *H. contortus* interaction with the host is expected to identify new drug targets for controlling *H. contortus* infection in the future.

*H. contortus* is one of the most important parasitic nematodes of considerable economic importance in small ruminants such as sheep and goats (6). In the host–parasite relationship, the parasite excretes and secretes a large number of molecules into the host to regulate the host’s immune function. It was shown that *H. contortus* excretory/secretory products (HcESPs) coincubated with PBMCs *in vitro* inhibited the production of IL-4 and IFN-γ, suppressed cell proliferation and nitric oxide secretion, and promoted IL-10 secretion for anti-inflammatory effects (7). A variety of molecules derived from *H. contortus* nematodes have been identified to modulate the function of PBMCs, such as rHc-STP-1 (8) and rHc-TpMy (9), which have unique immunosuppressive effects on goat PBMCs and may be one of the mechanisms that promote immune evasion.

TPS catalyzes the first step in trehalose synthesis (10), which involves the transfer of glucose from uridine diphosphate glucose (UDPG) to glucose 6-phosphate (G6P) to form trehalose-6-phosphate (11). Recent studies have shown that TPS is also involved in the regulation of insect physiology and behavior, including survival, molting, pupal metamorphosis, and chitin metabolism (12–15). In cotton bollworms, it was shown that TPS expression levels were higher in the nymphal stagnation period than in the non-stagnation period and could resist cold during the nymphal stagnation of cotton bollworms (16, 17). Trehalose, catalyzed by TPS, is an important defense mechanism for many pathogens, especially those living in extreme environments (18). Studies have shown that trehalose has specific bioprotective properties that protect organisms in harsh environments (19–24), including drying, dehydration, high acidity, heating, freezing, and oxidation. In nematodes, the concentration of trehalose is usually higher than that of free glucose, which provides energy as the main circulating sugar and is important for egg incubation (25). However, little is known about the role TPS plays in the growth, development, metabolism, and parasitism of *H. contortus*.

This study identifies the transcription of Hc-TPS protein in different developmental phases of the *H. contortus* and localization of expression in the adult stage. In addition, the effect of rHc-TPS on the function of goat PBMCs was assessed.

## Materials and Methods

### Ethics Declaration

Animal experiments were conducted following the guidelines of the Animal Ethics Committee, Nanjing Agricultural University, China. All experimental rules were approved by the Science and Technology Agency of Jiangsu Province. The approval ID is SYXK (SU) 2017-0027.

### Animals, Parasites, and PBMC Isolation

Three-to-six-month-old native crossbred goats from the Nanjing Agricultural University research and teaching herd were housed indoors and provided with microbial-free feed with free drinking water. The parasites (*H. contortus*) were maintained through consecutive passages of worm-free goat. Eggs, third-stage larvae (L3s), exsheathed L3s (xL3s), adult males, and adult females were collected as mentioned previously (7, 26). Healthy goats were kept individually in ventilated cages to prevent accidental infection from nematodes and fed them with hay, whole–shell corn, and free freshwater in their enclosures. The blood samples were taken from healthy goats. Afterward, PBMCs were assembled by the gradient centrifugation technique (27).

Female Wistar rats (body weight 250 g) were procured by the Animal Experimental Station of Jiangsu and raised in the Nanjing Agricultural University Experimental Animal Center.

### Cloning of Hc-TPS and Expression of rHc-TPS

*H. contortus* complementary DNA (cDNA) was synthesized as previously described (28). The 160–702-aa region of the glycosyltransferase domain-containing protein (GenBank: HF965754.1) is the 6-phosphorylase synthase structural domain (Hc-TPS). The nucleotides 160 to 702 aa of the glycosyltransferase domain-containing protein were cloned using specific primers (**Supplementary Table 1**). The PCR amplification was carried out using 2 × Phanta Master Mix (Vazyme Biotech, Nanjing, Jiangsu, China). The cyclic terms were used accordingly: the first denaturation for 3 min at 94°C (1 cycle), denaturing (10 s at 94°C), annealing (30 s at 65°C), extension (2 min at 72°C) (30 cycles), and final extension (72°C for 5 min (1 cycle). The PCR product was cleaned by using Gel Extraction Kit (Vazyme Biotech, Nanjing, Jiangsu, China). The Hc-TPS gene was cloned into the pET28a (+) vector (Novagen, Madison, WI, USA) expression plasmid with *Sac* I*/Xho* I restriction sites. Then, the Hc-TPS gene was again sequenced to confirm the right reading frame. The recombinant plasmid (pET28a (+) ligated with Hc-TPS) was again cultured into LB (Luria-Bertani) media used with kanamycin sulfate (optical density at OD600), and the expression was induced into *E. coli* (BL21, DE3) *via* isopropyl-β-D-thiogalactopyranoside (IPTG; working concentration: 1 mM) for 5 h at 37°C (29). The rHc-TPS protein was attached with a histidine-tag and obtained from bacterial lysis *via* His-Trap HP columns (GE Healthcare, Piscataway, NJ, USA), and dialysis was performed by renaturation buffer (20 mmol/l Tris–HCl, 500 mmol/l NaCl, 1 mmol/l GSH, 0.1 mmol/l GSSG, pH 8.0) containing different urea concentrations (8, 6, 4, 2, and 0 M) and using PBS (pH 8.0). The rHc-TPS protein was determined (size and purity) on 12% sodium dodecyl sulfate-polyacrylamide gel electrophoresis (SDS-PAGE). The lipopolysaccharide (LPS) was decontaminated with the rHc-TPS protein by the use of the Endotoxin Removal Kit (GenScript, Nanjing, China), and the concentration was measured by using the BCA kit (Thermo Fisher Scientific, Rockford, USA).

### Preparation of Polyclonal Antibodies

The polyclonal antibodies were generated against rHc-TPS. Approximately 300 µg of rHc-TPS protein was mixed 1:1 with Freund’s complete adjuvant (Sigma-Aldrich, Shanghai, China) and injected subcutaneously on multiple sites in the Wistar rats. Two weeks later, the rats received three booster doses of the same concentrations of protein with Freund’s incomplete adjuvant (1:1, Sigma-Aldrich, Shanghai, China), each 7 days apart. Seven days after the latter booster immunization, rats were anesthetized and anti-rHcTPS antibodies were collected in the serum.

### Detection of rHc-TPS by Western Blot Assay

The rHc-TPS protein (30 µg) was separated by 12% SDS-PAGE and transferred to polyvinylidene difluoride (PVDF) membranes (Millipore, USA) as described previously (30). Afterward, the non-specific binding site by skim milk (5%) in Tris-buffered saline solution consisting of 0.1% Tween-20 solution (TBST) was blocked. Subsequently, the membranes were cleaned three times by TBST and incubated with a primary antibody (serum from goat infected with *H. contortus*, 1:100 dilution in TBST) at 4°C overnight. The membranes were again cleaned and incubated by HRP-conjugated rabbit anti-goat IgG (diluted 1:5,000, USA) for 1 h at 37°C. Finally, the blots were revealed by enhanced chemiluminescence (ECL) in an ImageQuant 300 cabinet (GE Healthcare Biosciences, USA) according to the manufacturer’s instructions.

### Evaluating Transcript Abundance Using qPCR

Transcription of Hc-TPS was observed in different developmental stages (eggs, L3s, xL3s, and adults) of *H. contortus* by qPCR using the primers (HcTPS-F and Hc-TPS-R) (**Supplementary Table 2**). Total RNA was separated from eggs, L3s, xL3s, female adults, and male adults used by the TRIzol method (Vazyme Biotech, Nanjing, Jiangsu, China) according to the manufacturer’s protocol. The cDNA was synthesized by the HiScript III 1st Strand cDNA Synthesis Kit (Vazyme Biotech, Nanjing, Jiangsu, China). The β-tubulin gene was utilized as the referring gene. The data were investigated according to raw cycle thresholds (Ct) which were attained from ABI Prism 7500 software (Applied Biosystems, USA) used by the relative Ct (2^-ΔΔCt^) method. Three separate experiments were carried out with the replicates of each group.

### Localization of Hc-TPS in Mature Worms (*H. contortus*)

The adult worms were collected from goat abomasum and placed immediately for fixation into a solution of 4% paraformaldehyde solution for 12 h. Samples were embedded into paraffin wax, and worms (*H. contortus*) were sliced into 4-µm-thick sections using a rotary slicer (Leica, Germany). The slides were processed with citrate buffer (0.01 mol/l) to repair the antigen, and then non-specific binding was blocked with 5% skim milk for 1 h at room temperature. Sections were incubated with primary antibody (rat-anti-rHcTPS antiserum and normal rat serum) at 4°C overnight. Subsequently, three times washed with PBS and incubated with a Cy3-coupled secondary antibody (goat-anti-rat IgG) for 1 h at 37°C. The 4′,6-diamidino-2-phenylindole (DAPI, Beyotime, Nanjing, China) was used to stain the corresponding nuclei within the worm sections, incubated for 7 min, and washed again three times with PBST. In the end, the longitudinal cross section was observed using a laser confocal microscope (LSM 710, Zeiss, Germany).

### Binding of rHc-TPS to Goat PBMCs

Freshly isolated goat PBMCs were incubated with rHc-TPS protein (10 µg/ml) and the same volumes of control buffer (PBS) for 1 h at 37°C, respectively. Briefly, cells were fixed in 4% paraformaldehyde on polylysine-coated glass slides and blocked *via* skim milk (5%) for 30 min at room condition. Afterward, the slides were incubated with rat anti-rHc-TPS serum (1:100) at 4°C overnight, cleaned three times with PBS, and again incubated with Cy3-tagged goat anti-rat IgG (1:500) at 37°C for 1 h. Prior to staining with DAPI (Beyotime, Nanjing, China), the nuclei were again washed, and cells were imaged using a confocal laser scanning microscope (LSM 710, Zeiss, Germany).

### Cell Proliferation Assay

PBMCs (1 × 10^6^ cells/ml) were incubated with serial concentrations of rHc-TPS (10, 20, 40, and 80 µg/ml) and the same volume of control buffer (PBS) with 5% CO_2_ for 24 h at 37°C. The cell proliferation assays were detected by adding 10 μl of CCK-8 solution (Beyotime, Nanjing, China) with another 2-h incubation, after which the OD_450_ (optical density) values were calculated by using a microplate reader (Thermo Scientific, USA). The OD_450_ value of the control was set at 100%, and the following equation was used to calculate the cell proliferation index: OD_450_ sample/OD_450_ control. Three independent trials were conducted with three technical repeats of each group.

### Nitric Oxide Production Assay

PBMCs (100 μl, 1 × 10^6^ cells/ml) were inoculated in a DMEM culture medium in 96-well plates. The cells were incubated with rHc-TPS (10, 20, 40, and 80 µg/ml) and PBS as a control (at 37°C and 5% CO_2_ for 24 h). The subcellular nitric oxide generation of PBMCs was calculated using a Nitric Oxide Assay Kit (Beyotime, Shanghai, China). Three independent trials were conducted with repeats per group.

### Transcriptional Abundance of Inducible Nitric Oxide Synthase, Cytokines, and Related Pathway Molecules Detected by qPCR

Group settings were consistent with the description of nitric oxide production assay. Total RNA extraction and cDNA preparation were performed as previously reported (31). The primers INF-γ, IL-2, IL-4, IL-9, IL-10, IL-17, transforming growth factor β (TGF-β), IL-2R, inducible nitric oxide synthase (iNOS), and signal transducer and activator of transcription 5 (STAT5) for qPCR are listed in **Supplementary Table 2**. The β-actin gene was utilized as a reference gene. The data were calculated according to raw cycle thresholds (Ct) which were attained from ABI Prism 7500 software (Applied Biosystems, USA) using the relative Ct (2^-ΔΔCt^) method. Three independent trials were conducted for each group.

### The Expression Abundance of IL-10, p-STAT3, and SOCS3 Protein Detection by Western Blot Assay

Group settings were consistent with the description of nitric oxide production assay. Total protein was extracted after washing the PBMCs with PBS, and 70 μl of RIPA modified lysis buffer was added (Beyotime, Nanjing, China). Protein concentrations were determined through the Bradford method used by Bio-Rad Protein Assay (Bio-Rad, USA) reagent and bovine serum albumin (BSA, Sigma-Aldrich Co.) as a standard. Proteins (30 µg) were transferred to polyvinylidene difluoride (PVDF) membranes (Millipore, USA) for Western blot analysis. The non-specific binding was blocked with 5% BSA. The membranes were cleaned three times *via* TBST and then incubated with first antibodies (anti-p (Tyr705)-STAT3 (Novus Biologicals, USA), anti-SOCS3 (Beyotime, Nanjing, China), anti-IL-10 (Affinity Biosciences, china), and anti-β-actin (AB clonal, China)) overnight at 4°C. The membranes were again washed three times and incubated with HRP-conjugated goat anti-rabbit IgG (Beyotime, China) at 37°C for 1 h. Finally, the blots were revealed by enhanced chemiluminescence (ECL) in an ImageQuant 300 cabinet (GE Healthcare Biosciences, USA) according to the manufacturer’s instructions. The band intensity was analyzed through ImageJ software.

### Detection of Nuclear Translocation of STAT3 by IFA

Group settings were consistent with the description of nitric oxide production assay. IFA used the STAT3 polyclonal antibody (1:50, Affinity Biosciences, China) as the primary antibody and goat anti-rabbit IgG coupled to Cy3 as the secondary antibody. This was performed with reference to the binding of rHc-TPS to goat PBMC assay.

### Data Analysis

Statistical analyses were performed by using the GraphPad Premier 6.0 software package (GraphPad Prism). The results were presented in terms of mean ± SEM. The Student’s t-test was performed to determine differences between the two groups. The differences between the groups were statistically calculated by one-way analysis of variance (ANOVA). *p*-value < 0.05 was considered as statistically significant.

## Results

### Cloning, Expression, and Western Blot Analysis of Hc-TPS

**Figure 1:**
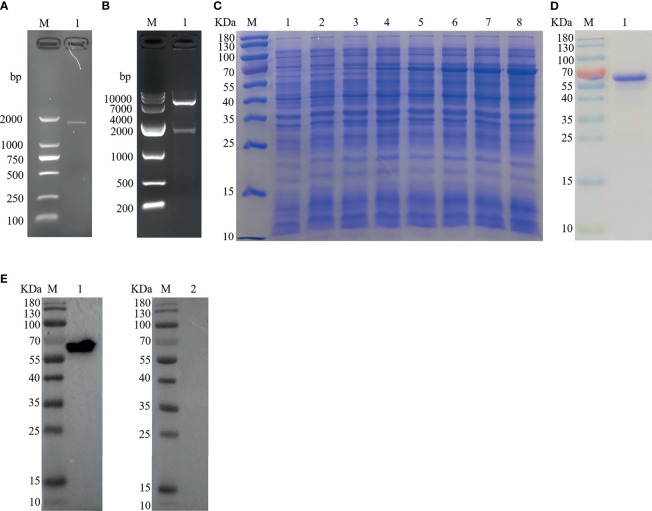
Cloning, expression, and Western blottin analysis of Hc-TPS. **(A)** Amplification of the Hc-TPS gene. Lane M: DNA Marker DL 2000; Lane 1: the amplification products of TPS gene. **(B)** Lane M: DNA Marker DL10000; Lane 1: digestion of pET-28a/Hc-TPS by enzymes. **(C)** The expression of Hc-TPS. Lane M: standard protein molecular weight marker; Lane 1~2: pET-28a induced by IPTG for 0 and 5 h; Lane 3~8: pET-28a/Hc-TPS induced by IPTG for 0~5 (h) **(D)** Lane M: standard protein molecular weight marker; Lane 1: purification of rHc-TPS. **(E)** Western blot examination of rHc-TPS. Lane M: standard protein molecular weight marker; Lane 1: rHc-TPS detected by serum from *H. contortus* experimentally infected goat; Lane 2: reaction with normal goat sera.

The PCR product of Hc-TPS amplification was attained from *H. contortus* by using cDNA with a precise set of primers, and the right segment size of 1,629 bp (**Figure 1A**) of the 543 protein-encoding amino acids was detected. The Hc-TPS gene was effectively cloned into the pET-28a expression vector, and 1,629 bp was verified by enzymatic digestion *via* online BLAST analysis (**Figure 1B**). The recombinant plasmid (pET-28a/Hc-TPS) was induced *via* IPTG and expressed in *E. coli* BL21 (DE3), and rHc-TPS protein was displayed on SDS-PAGE with Coomassie blue staining (**Figure 1C**). Purified rHc-TPS was obtained using Ni2+ affinity chromatography, and a single band with a predetermined fraction of 64 kDa was identified (**Figure 1D**). Moreover, the results of the Western blot showed that rHc-TPS protein was recognized by the goat serum infected with *H. contortus* but could not be recognized by the sera from normal goat (**Figure 1E**). This suggests that Hc-TPS may have potential as a diagnostic antigen or modulator of host immune function.

### Localization of Hc-TPS in Adults and Transcription at Different Developmental Stages of *H. contortus*

**Figure 2:**
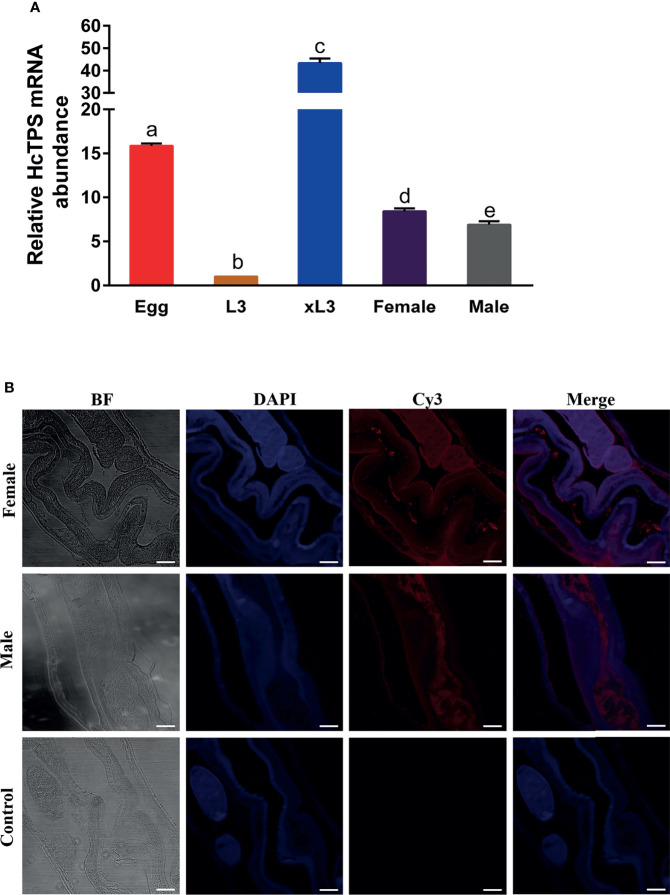
Localization of Hc-TPS in adults and transcription at different stages of *H*. *contortus*. **(A)** Transcriptional analysis of the Hc-TPS gene in different developmental phases of *H.contortus*. The relative quantities (compared with L3, L3 = 1) are shown as mean values. The results showed here a representative of three independent experiments, and data are represented as mean ± SEM. The Student’s t-test was performed to determine differences between the two groups; values without the same letter (a–e) are significantly different *(p* < 0.05). **(B)** Localization of Hc-TPS in adult (male/female) *H. contortus*. Worm sections were incubated with rat-anti-rHcTPS serum and Cy3-coupled goat-anti-rat IgG. DAPI was used to stain the corresponding nuclei. The red color indicates the localization of target protein (Cy3), and the blue color indicates the localization of nuclei (DAPI). No red color was observed in the control (scale-bars: 50 μm).

The transcription of the Hc-TPS gene at various developmental stages of *H. contortus* was examined by RT-qPCR assay, and the β-tubulin gene was used as an internal reference. As shown in **Figure 2A**, Hc-TPS transcript levels were the lowest in the L3 stage and significantly higher in the adult (female and male), xL3, and egg stages compared with the L3 stage, especially in the egg and xL3 stages. In the adult stage, Hc-TPS transcription was significantly increased in females when compared with males. Furthermore, the longitudinal sections of adult male and female *H. contortus* worms were used in IFA to identify the localization of Hc-TPS protein. As shown in **Figure 2B**, Hc-TPS showed a wide distribution in the worm, especially high expression in the intestine of the worm as well as in the embryo of the female (some IFA data not shown).

### Effects of rHc-TPS on Different Functions of Goat PBMCs

**Figure 3:**
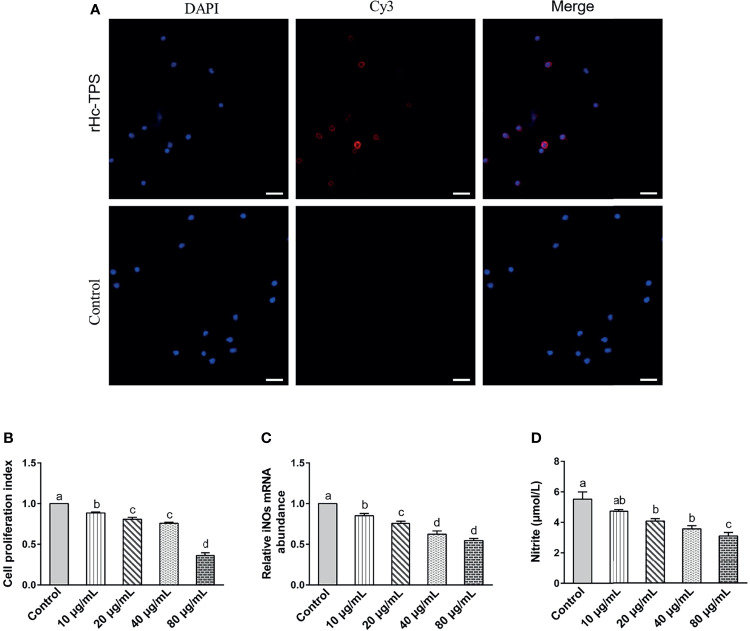
Effects of rHc-TPS on different functions of goat PBMCs. **(A)** Binding of the rHc-TPS protein to PBMCs. rHc-TPS protein was incubated with goat PBMCs, and the binding was detected by rat anti-rHc-TPS serum and Cy3-labelled goat anti-rat IgG. DAPI was used to stain the corresponding nuclei of PBMCs. No red color was observed in the control (scale bars: 20 μm). **(B)** Impacts of rHc-TPS on PBMC proliferation. Cells were incubated *via* various doses of rHc-TPS protein, PBS (control), and the cell proliferation index was calculated by setting the OD_450_ values with the control group as 100%. **(C, D)** Effect of rHc-TPS on nitric oxide production and iNOS transcription by PBMCs *in vitro*. Cells were incubated with serial concentrations of rHc-TPS protein or PBS (control) for 24 h at 37°C and 5% CO_2_. Data are presented as the mean ± SEM from three independent experiments, with three technical replicates per group. “a, b, c, or d” based one-way ANOVA analysis to indicate the significance, values without the same letter (a–d) are significantly different *(p* < 0.05).

The binding of recombinant proteins to specific receptors of PBMCs is an important way for them to exert immuno-modulation (32, 33). Previous studies in our laboratory have shown that His-tag could not affect the binding of recombinant proteins to PBMCs (31, 34, 35). The binding of rHc-TPS to goat PBMCs was confirmed by IFA. The results showed that the rHc-TPS protein can attach to the cell surface (**Figure 3A**). Moreover, the impact of the rHc-TPS protein on cell proliferation was detected *via* the Cell Counting Assay Kit 8 (CCK8). The proliferation of PBMCs incubated with various doses (10, 20, 40, and 80 μg/ml) of rHc-TPS protein was considerably restrained when compared with the control group (**Figure 3B**). Furthermore, the effects of rHC-TPS on transcript levels of iNOs and NO secretion in PBMCS were examined by qPCR assay and nitric oxide assay kit, respectively. Compared with the control group, different concentrations of rHc-TPS significantly inhibited the transcription of iNOS in PBMCs and showed a dose-dependent relationship (**Figure 3C**). Consistent with expectations, rHC-TPS inhibited NO secretion from PBMC cells in a dose-dependent manner (**Figure 3D**).

### Effect of rHc-TPS Protein on the Transcription of Cytokines in PBMCs

**Figure 4:**
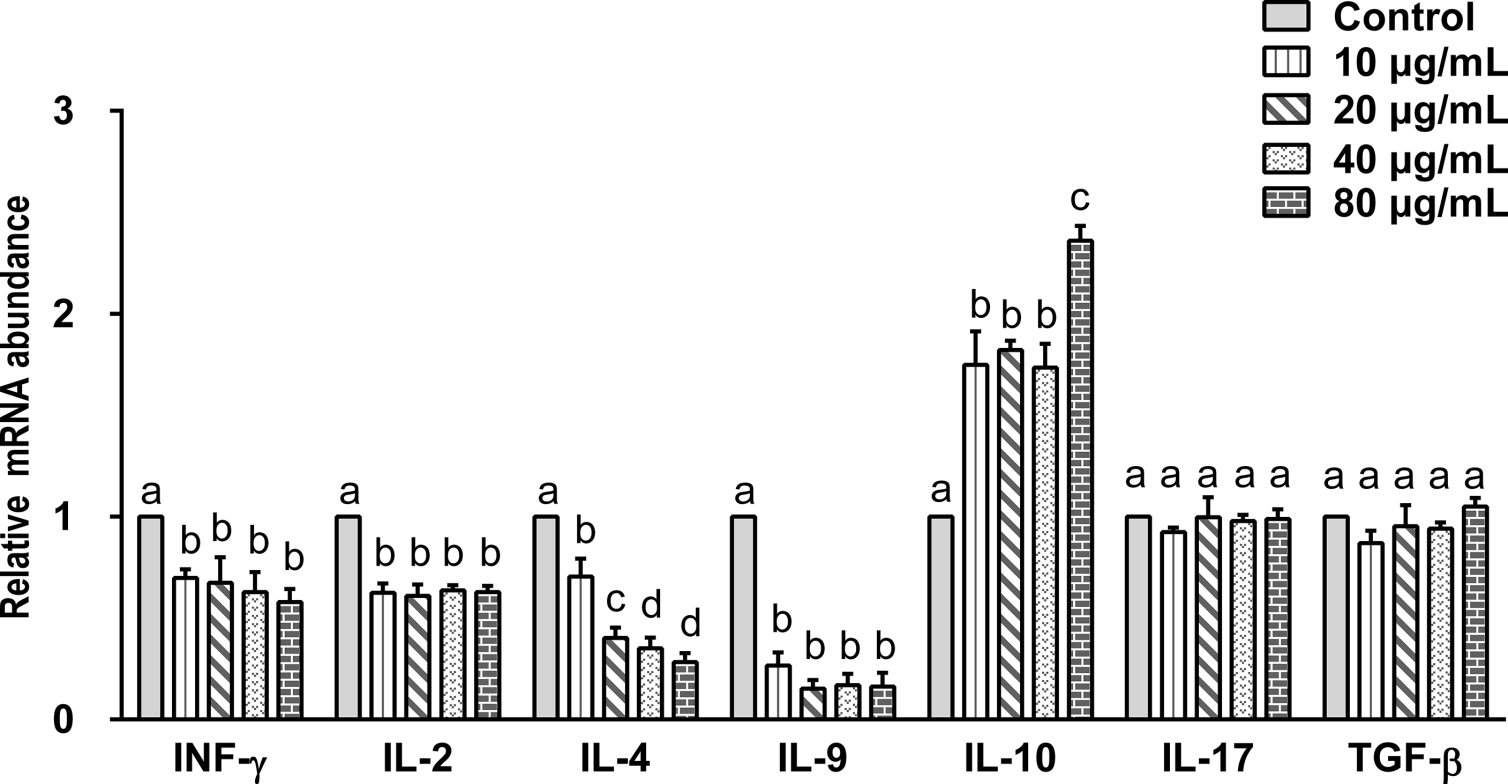
Impact of rHc-TPS protein on the transcription of cytokines in PBMCs. Cells were incubated with serial concentrations of rHc-TPS protein or PBS (control) for 24 h at 37°C and 5% CO_2_. The transcription of INF-γ, IL-2, IL-4, IL-9, IL-10, IL-17, and TGF-β were tested by qPCR. The data were presented of three independent trials (mean ± SEM). “a, b, c, or d” based one-way ANOVA analysis to indicate the significance; values without the same letter (a–d) are significantly different *(p* < 0.05).

Data indicated that there are seven main types of immune responses mediated by T helper cells (Th), including Th1, Th2, Th17, Treg, Tfh, Th9, and Th22 (36). Studies on the immune mechanism of *H. contortus* have focused on Th1 (INF-γ, IL-2), Th2 (IL-4), Th9 (IL-9), Treg (IL-10, TGF-β), and Th17 (IL-17) immune responses. Our aim was to detect the effect of rHc-TPS on cytokines associated with these immune types. The effect of rHc-TPS protein on cytokine production in PBMCs was examined by qPCR assay, which showed that the transcript levels of INF-γ, IL-2, IL-4, and IL-9 were substantially reduced compared with the control group. In contrast, rHc-TPS significantly promoted the transcription level of IL-10 mRNA in PBMCs. However, different concentrations of rHc-TPS had no significant effect on the transcription of IL-17 and TGF-β in PBMCs (**Figure 4**).

### Effect of rHc-TPS Protein on the Transcription of the IL-2/STAT5 Signal Pathway

**Figure 5:**
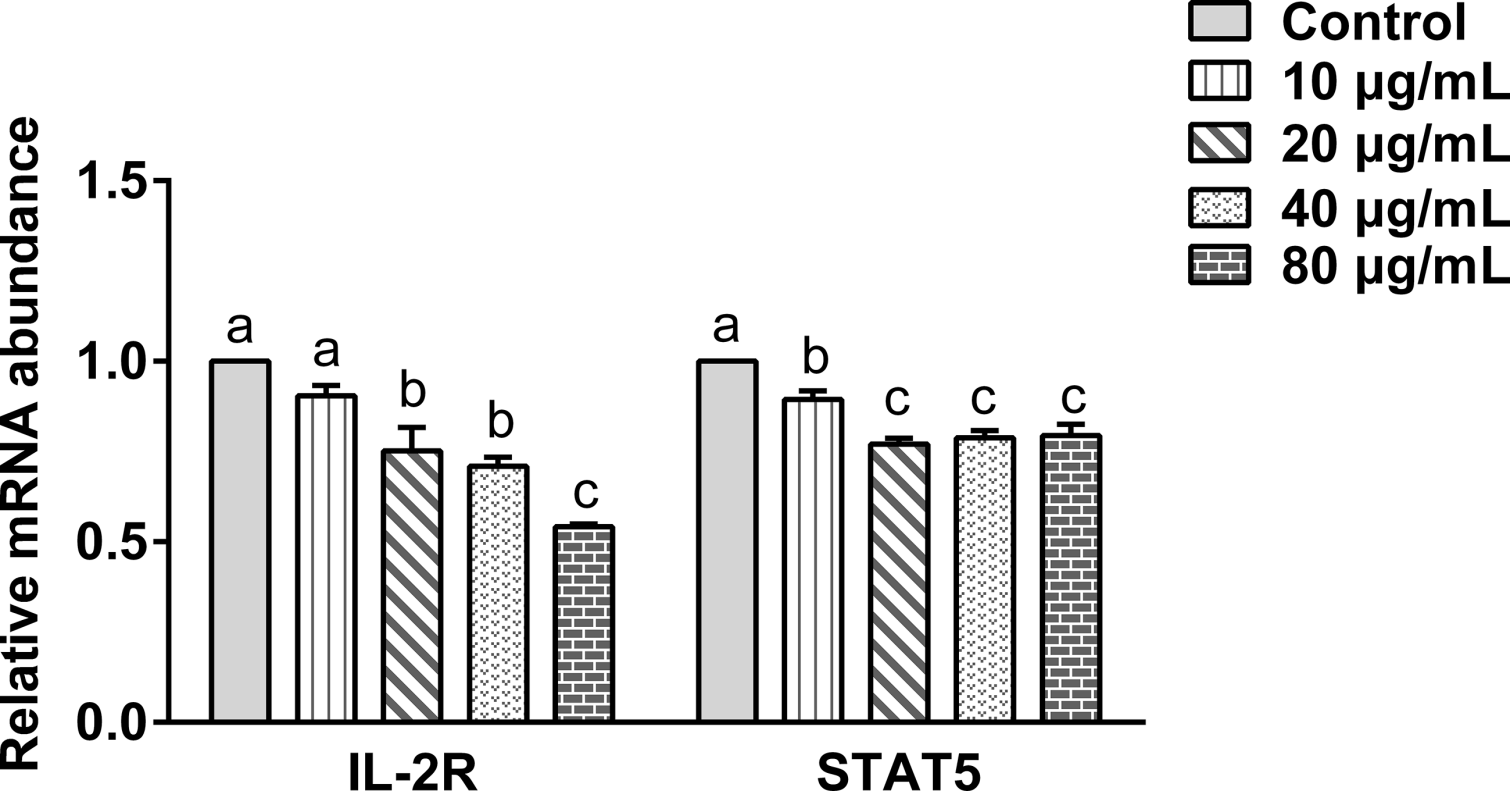
Effect of rHc-TPS protein on the transcription of the IL-2/STAT5 signaling pathway. Cells were incubated with serial concentrations of rHc-TPS protein or PBS (control) for 24 h at 37°C and 5% CO_2_. The data were presented of three independent trials (mean ± SEM). “a, b, or c” based one-way ANOVA analysis to indicate the significance; values without the same letter (a–c) are significantly different *(p* < 0.05).

The effect of the rHc-TPS coculture with PBMCs on IL2-R and STAT5 gene transcription was analyzed by qPCR. **Figure 5** displays the effect of rHc-TPS protein on the IL-2/STAT5 pathway, and the result showed that transcript levels of IL-2R and STAT5 were significantly reduced.

### The rHc-TPS Protein Activated the IL-10/STAT3/SOCS3 Signaling in PBMCs

**Figure 6:**
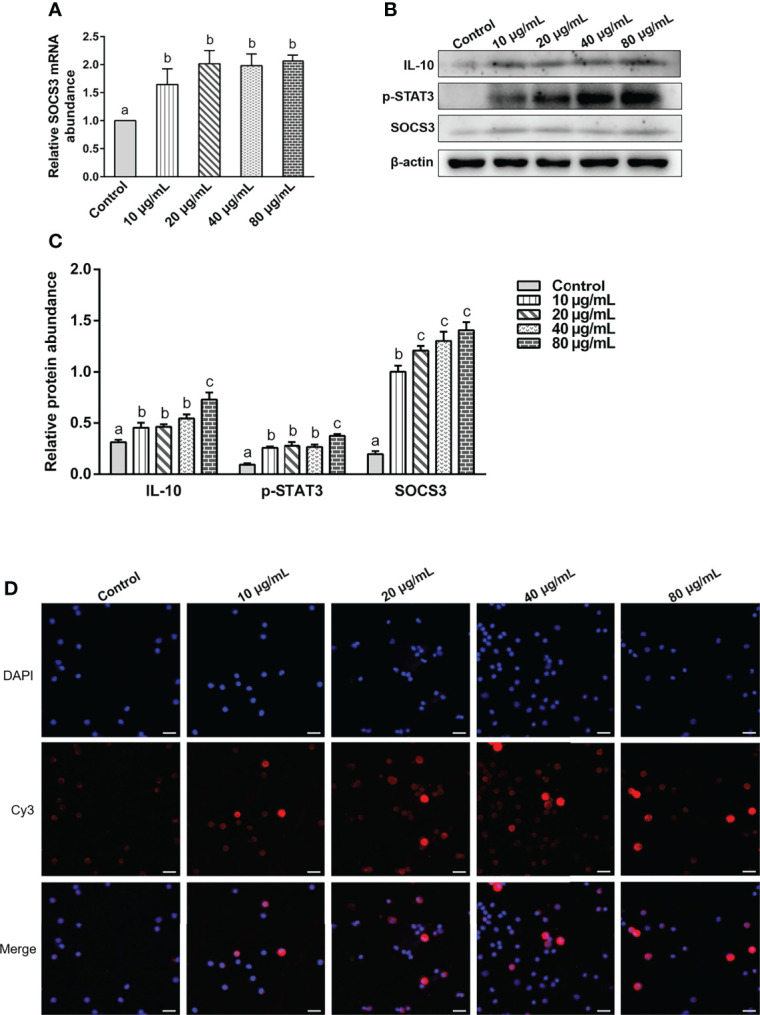
The rHc-TPS protein activated the IL-10/STAT3/SOCS3 signaling in PBMCs. Cells were incubated with serial concentrations of rHc-TPS protein or PBS (control) for 24 h at 37°C and 5% CO_2_. **(A)** Transcription study of SOCS3 in goat PBMCs. **(B)** The expression of IL-10, p705-STAT3, and SOCS3 were detected by Western blot. **(C)** Statistics of the IL-10, p705-STAT3, and SOCS3 Western blotting results. The data were presented of three independent trials (mean ± SEM). “a, b, or c” based one-way ANOVA analysis to indicate the significance, values without the same letter (a–c) are significantly different (*p* < 0.05). **(D)** Detection of nuclear translocation of phosphorylated STAT3 by IFA; the red color shows the specific protein localization stained *via* Cy3 on PBMCs, and the blue color indicates the nuclei *via* DAPI. Merged picture shows both DAPI and Cy3. Scale bars: 20 μm.

Various concentrations of rHc-TPS protein remarkably promoted the transcription of SOCS3 in PBMCs (**Figure 6A**). It also promoted the expression levels of IL-10, p(Tyr705)-STAT3, and SOCS3 in PBMCs (**Figures 6B, C**). IFA results showed that rHc-TPS promotes STAT3 to undergo nuclear translocation (**Figure 6D**).

## Discussion

Recent studies have shown that TPS plays an important role in invertebrate growth and development, stress recovery, energy metabolism, chitin synthesis, molting, and other biological processes (11). TPS and trehalose 6-phosphate phosphatase (T6PP) together catalyze the biosynthesis of trehalose, while their synthesis pathways are lacking in mammals. These are promising targets for the development of antibacterial, antifungal, and anthelmintic therapies (37). Our study found that Hc-TPS was transcribed at different developmental phases of *H. contortus*, with relatively high transcript levels in eggs as well as exsheathed L3. In addition, IFA results showed that Hc-TPS was widely distributed in adults, with localized expression mainly on the gut surface and in the embryos of females. TPS transcriptional expression in *H. contortus* was similar to that of trehalose 6-phosphate phosphatase (GOB, unpublished results). The high Hc-TPS gene expression in females as compared to males might be related to the high Hc-TPS expression in the embryo. This implies that we need to further investigate whether Hc-TPS gene silencing or antibody sequestration of the Hc-TPS protein has important effects on larval molting and embryonic development. Western blot results revealed that rHc*-*TPS protein was identified by sera of goats infected with *H. contortus*, suggesting that Hc-TPS can be identified by the host immune system. In addition, the immunolocalization of Hc-TPS was similar to the *Barbervax^®^* antigens H-gal-GP and H11 (38, 39), which were locally expressed on the intestinal mucosal surface of *H. contortus*, suggesting that Hc-TPS was more susceptible to attack by host peripheral blood circulating antibodies and may be a vaccine candidate.

When stimulated by antigens, naïve T cells were activated by antigen-presenting cells, and adaptive immune responses were stimulated by cytokine secretion and cell proliferation (40). It has been shown that the ESP of *Brugia malayi* inhibit the proliferation of T lymphocytes, which may contribute to the inability in eliminating the parasite (41–43). In the present study, rHc-TPS protein was found to significantly inhibit the proliferation of PBMCs and showed a dose-dependent manner. This indicated that Hc-TPS could be a suppressive antigen of goat PBMCs. Our results were consistent with previous studies (7, 9). NOS catalyzes the creation of NO, citrulline from L-arginine, oxygen, and cofactors. NO is the product of activation of immune cells by cytokines, microorganisms, parasites, etc. (44). It acts as an antibacterial, antitumor, and parasiticidal agent studied *in vitro* as well as *in vivo* (45, 46). Our study showed that rHc-TPS significantly inhibited the transcription of iNOs and the secretion of NO in PBMCs.

Previous studies showed that Th1, Th2, Th9, and Th17 immune responses play important roles in fighting parasitic infections (47–50). Our results showed that rHc-TPS co-incubation with PBMCs significantly inhibited the transcriptional levels of IFN-γ, IL-4, and IL-9, which may exert one of the pathways of immune evasion. IL-2 produced by antigen or mitogen-stimulated helper T-cells was one of the most indispensable immunomodulatory factors in immune regulation (51, 52). IL-2 signaling acts through STAT5 and affects the differentiation of helper T-cell subsets (including Th1, Th2, Th9, and Th17 cells), as well as Treg cells (51). IL-2/STAT5 positively regulates the differentiation of Th1, Th2, and Th9 cells; however, it exerts a negative regulatory effect on the differentiation of Th17 cells. In the present study, rHc-TPS was found to significantly inhibit the IL-2/STAT5 pathway, as described in previous studies (53). Thus, rHc-TPS inhibited the transcription of IFN-γ, IL-4, and IL-9 in PBMCs cells, possibly acting through inhibition of the IL-2/STAT5 axis.

IL-10 is a cytokine with anti-inflammatory properties that plays a central role in infection by limiting the immune response to pathogens and preventing the host from overactive inflammatory responses (54). STAT3 is a key effector molecule for IL-10 activation and results in upregulation of SOCS3, which is necessary for IL-10-regulated anti-inflammatory effects (55–59). Our study found that co-incubation of the Hc-TPS protein with PBMCs significantly promoted the transcriptional expression of the anti-inflammatory factor IL-10. Further studies revealed that rHc-TPS significantly activated STAT3, thereby promoting the transcriptional expression of SOCS3. Activation of the IL-10/STAT3/SOCS3 axis by rHc-TPS may be necessary for immune evasion in *H. contortus*.

## Conclusions

In conclusion, the Hc-TPS gene was transcribed at high levels in egg and xL3 stages and was highly expressed in the gut of the adult stage. rHc-TPS could activate the IL-10/STAT3/SOCS3 axis to exert anti-inflammatory effects, and inhibit the proliferation of PBMCs and the secretion of the pro-inflammatory factor NO, while suppressing the transcription of IL-2, IL-4, INF-γ, and IL-9. However, further studies are required to examine the potency of rHc-TPS protein in the goat infection models. Our findings could assist to provide insight into the molecular mechanisms of this protein under host–parasite interactions.

## Data Availability Statement

The original contributions presented in the study are included in the article/**Supplementary Material**. Further inquiries can be directed to the corresponding author.

## Ethics Statement

Animal experiments were conducted following the guidelines of the Animal Ethics Committee, Nanjing Agricultural University, China. All experimental rules were approved by the Science and Technology Agency of Jiangsu Province. The approval ID is SYXK (SU) 2017-0027.

## Author Contributions

Data curation, ZW and MA. Formal analysis, ZW and MA. Funding acquisition, RY. Investigation, KA. Methodology, ZW, CC. Project administration, RY. Resources, ZW and MA. Software, ZW and KA. Supervision, XS, LX, XL, and RY. Visualization, ML. Writing—original draft, ZW. Writing—review and editing, ZW, MA, and RY. All authors contributed to the article and approved the submitted version.

## Funding

This research was funded by the National Natural Science Foundation of China (31872464), the policy guidance project of Jiangsu Province for international scientific and technological cooperation (BZ2019013), the National Key Research and Development Program of China (2017YFD0501200), and the Priority Academic Program Development of Jiangsu Higher Education Institutions (PAPD).

## Conflict of Interest

The authors declare that the research was conducted in the absence of any commercial or financial relationships that could be construed as a potential conflict of interest.

## Publisher’s Note

All claims expressed in this article are solely those of the authors and do not necessarily represent those of their affiliated organizations, or those of the publisher, the editors and the reviewers. Any product that may be evaluated in this article, or claim that may be made by its manufacturer, is not guaranteed or endorsed by the publisher.

## Abbreviations

GT1-TPS, trehalose phosphate synthase structural domain protein; LB, Luria Bertani; IPTG, isopropyl-β-D-thiogalactopyranoside; Ni-NTA, Ni2+-nitrilotriacetic acid; PVDF, polyvinyl difluoride; TBS-T, Tris-buffered saline containing 0.05% Tween-20; CCK-8, cell counting kit-8; NO, nitric oxide; qPCR, quantitative real-time PCR; IFA, immunofluorescence assay; PBS, phosphate-buffered saline; Th, CD4+ T helper cells; IFN-γ, interferon gamma; IL, interleukin; STAT5, signal transducer and activator of transcription 5; SOCS3, suppressors of cytokine signaling 3; STAT3, signal transducer and activator of transcription 3.
