# Supplementary material for: The GT1-TPS Structural Domain Protein From Haemonchus contortus Could Be Suppressive Antigen of Goat PBMCs
Source: Front Immunol. 2022 Jan 4;12:787091. doi: 10.3389/fimmu.2021.787091 (PMC8764253; doi:10.3389/fimmu.2021.787091)
Supplement: Supplementary file 1 [file Table_1.docx]

**Table 1. Primers used for PCR Amplification experiments**

| **Target genes** | **Primer sequences (5’-3’)** |
| --- | --- |
| TPS | C**GAGCTC**agggtgatcaatgtgtcgaatgc |
|  | CC**CTCGAG**atcaaggaaagcagtgctccac |

**Table 2. Primers used for qPCR experiments**

| **Target genes** | **Primer sequences (5’-3’)** |
| --- | --- |
| β-actin | CACCACACCTTCTACAAC |
|  | TCTGGGTCATCTTCTCAC |
| IL-2 | CAAACGGTGCACCTACTTCA |
|  | AGCTTGAGGTTCTCGGGATT |
| IL-4  IL-9 | GTACCAGCCACTTCGTCCAT |
|  | GCTGCTGAGATTCCTGTCAA  GATGCGGCTGATTGTTT  CTCGTGCTCACTGTGGAGT |
| IL-10 | CCTTGTCGGAAATGATCCAG |
|  | AGGGCAGAAAACGATGACAG |
| IL-17 | TTGTAAAGGCAGGGGTCATC |
|  | GGTGGAGCGCTTGTGATAAT |
| TGF-β | GAACTGCTGTGTTCGTCAGC |
|  | TCCAGGCTCCAGATGTAAGG |
| IFN-γ | GAACGGCAGCTCTGAGAAAC |
|  | GGTTAGATTTTGGCGACAGG |
| iNOS | TTTGCTCACGACATCGACCA |
|  | CTCAATGTGGTGCTTGCCAC |
| STAT5 | TTCCTTGCATTGCCCACACTCC |
|  | CAACACTCCACCCACCCTCAAAG |
| IL-2R  TPS | GGGCAGGCAGGAAAGTGTTA  GGGAATGCTGACGATGGAGT  TGGTCTGCCTCGTTCTCGTCTC  CCCTGCTCCCGATGAAAGTACAAG |
| β-tubulin | TGCTATGTTCCGTGGTCGTATG |
|  | CGGCAGTCTTAACGTTGTTTGG |
